# Increase in Ia Afferent Synaptic Excitation of SOD1 G93A Mouse Motoneurons by 2‐Week Anodal Trans‐Spinal Direct Current Stimulation Does Not Ameliorate the Cellular Burden of the Disease

**DOI:** 10.1111/ejn.70375

**Published:** 2025-12-25

**Authors:** T. Jankowiak, M. Cholewiński, K. Kryściak, E. Krok, K. Grycz, M. Bączyk

**Affiliations:** ^1^ Department of Neurobiology Poznań University of Physical Education Poznań Poland; ^2^ Faculty of Materials Engineering and Technical Physics, Department of Molecular Physics Poznan University of Technology Poznań Poland; ^3^ Université Paris Cité, Saints‐Pères Paris Institute for the Neurosciences Paris France

**Keywords:** electrophysiology, excitatory postsynaptic potentials, in vivo intracellular recordings, neurodegenerative disease, passive membrane properties

## Abstract

An imbalance between cells' intrinsic excitability and synaptic excitation levels underlies the spinal motoneuron (MN) pathophysiology in Amyotrophic Lateral Sclerosis. Recently, a transient restoration of the deficient Ia synaptic excitation of spinal MNs in the presymptomatic SOD1 G93A mice was achieved by applying a single trans‐spinal direct current stimulation (tsDCS) session. Here we investigate whether two‐week repeated tsDCS applied to presymptomatic SOD1 G93A animals can permanently alter spinal MN synaptic excitation levels and in this way affect intracellular metabolic pathways and cellular burden of the disease. Anodal, cathodal, or sham polarization of 100 μA was applied to P30‐P35 SOD1 G93A male mice, and passive membrane properties and Ia excitatory post‐synaptic potential (EPSP) characteristics were investigated by intracellular recordings of spinal MNs in vivo. A second cohort of animals was used to test the impact of our intervention on Ia synapse morphology, intracellular metabolic pathways activity, and disease markers. Anodal tsDCS evoked a strong increase in maximal Ia EPSPs amplitudes, coupled with a significant upregulation of GluR4 subunits of AMPA receptors at the Ia synapse. The cathodal polarization failed to induce any alteration to Ia synapse morphology, but increased the input resistance of MNs. However, changes in MN electrophysiological profile and Ia synapse morphology did not translate into alterations of intracellular molecular pathways activity and did not decrease the cellular burden of the disease. Our results indicate a strong polarity‐dependent plasticity of spinal MNs in SOD1 G93A mice in response to tsDCS, which however does not alleviate disease burden.

## Graphical abstract

In the presymptomatic SOD1 G93A mouse model of ALS, spinal motoneuron (MN) synaptic excitation is reduced due to disruptions in the postsynaptic elements of Ia synapses. Two weeks of anodal tsDCS were applied to SOD1 G93A animals to modulate MN synaptic excitation levels. The tsDCS intervention increased both the number and area of GluR4 subunits of AMPA receptors at VGluT1 synapses, resulting in enhanced MN synaptic excitation by proprioceptive afferents.

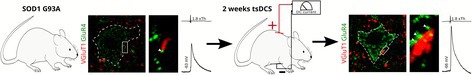

**Abbreviations**
ALS: amyotrophic lateral sclerosis
AMPA: α‐amino‐3‐hydroxy‐5‐methyl‐4‐isoxazolepropionic acid
CREB: cyclic AMP Response Element‐Binding Protein
CTB: cholera toxin B subunit
DC: direct current
DCC: discontinuous current clamp
ECG: electrocardiogram
EF: electric field
GluR4: glutamate subunit 4 of the AMPA receptor
Ia: large‐diameter, fast‐conducting nerve fibre innervating muscle spindles
Ih: hyperpolarization‐activated cationic current
Ih: hyperpolarization‐activated current
LG: the lateral gastrocnemius
misfSOD1: misfolded SOD1 protein
MN: motoneuron
P: postnatal day
PFA: paraformaldehyde
PIC: persistent inward current
PP: paired pulse protocol in which two successive stimuli are delivered to the peripheral nerve at a short 10 ms interval
RIN: input resistance
RMP: resting membrane potential
RQ: Relative quantification
SOD1 G93A: superoxide dismutase 1 with a glycine to alanine substitution at position 93
Th: threshold to activate the most excitable fibres in triceps surae nerve
TS: the triceps surae
tsDCS: trans‐spinal direct current stimulation
VGluT1: vesicular glutamate transporter 1
τ m: membrane time constant

## 1 Introduction

In trans‐spinal direct current stimulation (tsDCS) a low intensity DC is delivered through a pair of skin electrodes to modulate spinal activity via an induced electric field (Cogiamanian et al. 2008, 2012). Originally designed as a method to alleviate pain by blocking the corticospinal transmission of a nociceptive signal (Cogiamanian et al. 2011), tsDCS has evolved into a neuromodulation technique capable of affecting motor unit recruitment (Bocci et al. 2014) and providing functional gains both in physiological (Berry et al. 2017) and pathological conditions (Heide et al. 2014; Gómez‐Soriano et al. 2019; Zeng et al. 2020). Notably, tsDCS does not trigger neuronal activation, as in the case of spinal cord stimulation (SCS), (de Vos and Meier 2024) or transcutaneous electrical stimulation (TES), (Lieu et al. 2024). Attempts are now made to introduce tsDCS to the therapy of neurological disorders in which alterations of neuronal excitability may prove beneficial.

In Amyotrophic Lateral Sclerosis (ALS), a progressive loss of brainstem and spinal motoneurons (MNs) is classically linked to excessive calcium influx during abnormally high cell firing rates driven by elevated glutamate levels (Van Den Bosch et al. 2006; Ilieva et al. 2009). However, clinical trials aimed at decreasing MN firing in the hope of slowing their degeneration have never produced clinically relevant results (Petrov et al. 2017); the only exception is Riluzole treatment, which prolongs patients' survival in the terminal stage of the disease (Bensimon et al. 1994; Fang et al. 2018). On the other hand, chemogenetic interventions aimed at increasing the synaptic excitation of MNs have shown some positive effects on disease markers in animal ALS models (Saxena et al. 2013; Bączyk, Alami, et al. 2020). At this point, it is not clear if changes in excitability induce MN degeneration or reflect homeostatic mechanisms aimed at maintaining the firing rates (Elbasiouny 2022). However, it seems that interventions aimed at ameliorating the disease burden should be targeted toward restoring MN excitability and excitation profiles to physiological levels, depending on the disease stage. Recently, an article by Ahmed et al. (2025) demonstrated that long‐term application of anodal tsDCS to the SOD1 G93A mouse model reduces the levels of misfolded SOD1 protein in MNs and promotes mouse survival, highlighting the potential benefits of tsDCS in managing ALS. Interestingly, this finding directly contradicts the work of Highlander and Elbasiouny (Highlander and Elbasiouny 2022), who reported that chronic application of anodal tsDCS could be detrimental to the survival and functional scores of SOD1 G93A mice. However, neither study investigated the direct impact of tsDCS on MN activity, as no intracellular recordings of MN electrophysiological profiles were conducted. Therefore, the specific adaptations in the electrophysiological profiles of MNs due to tsDCS in ALS remain unclear.

Our own recent research showed that a single application of tsDCS temporarily altered the synaptic excitation levels of MNs in presymptomatic SOD1 G93A animals (Jankowiak et al. 2022). Specifically, we found that anodal (depolarizing) tsDCS increased the amplitudes of maximal monosynaptic Ia excitatory postsynaptic potentials (EPSPs) and raised the input resistance of spinal MNs. However, this effect was transient, returning to baseline levels within 2 h post‐application. In contrast, our experiments with wild‐type rats indicated that repeated tsDCS applications over several weeks led to significant neuroplastic changes in spinal MNs, reflected in alterations in their passive membrane properties and firing characteristics (Bączyk, Drzymała‐Celichowska, et al. 2020a). Building on these findings, our current study applied a two‐week tsDCS protocol to presymptomatic (P30‐P35) SOD1 G93A mice. We investigated the MN passive membrane properties, synaptic excitation levels, and Ia synapse morphology to assess the long‐term effects of tsDCS on the biophysical profile of MNs in SOD1 mice. We hypothesized that repetitive modulation of spinal excitability through tsDCS would result in measurable adaptations at both electrophysiological and morphological levels. Additionally, we expected that alterations in the synaptic excitation levels of ALS‐affected MNs would influence the activity of intracellular, activity‐dependent metabolic pathways, potentially impacting cellular burden of the disease. This mechanism could help clarify the positive outcomes observed by Ahmed et al. (2025). Our experimental results demonstrated that both anodal and cathodal tsDCS induced robust neuroplasticity in SOD1 G93A mouse spinal MNs. Anodal stimulation led to increased synaptic excitation, while cathodal stimulation altered the passive membrane properties of MNs. These effects were associated with polarity‐dependent changes in Ia synapse morphology, yet had minimal impact on intracellular pathway activity and the overall disease burden.

## 2 Materials and Methods

### 2.1 Animals

This study used a total of 48 male B6SJL‐Tg (SOD1*G93A)1Gur/J mice (SOD1 mice henceforth) bred at the Wielkopolska Center of Advanced Technologies at the Adam Mickiewicz University (Poznań, Poland). For all animals the gene's expression level was verified by comparing the relative quantification (RQ) value of each offspring to the RQ value of the transgenic father initially obtained from the Jackson Laboratory. Only mice with the RQ values in the range from 0.980 to 1.100 were selected for experiments. The RQ data were analyzed in StepOne Software v2.3.

We decided to limit our investigations to male mice to avoid sex‐related variability, as male SOD1 mice are characterized by more rapid disease progression when compared to female SOD1 mice (Oliván et al. 2015). Mice were housed two per cage at the Poznań University of Physical Education Animal Facility (Poznań, Poland) with unlimited access to food and water. The animal room was set to a reverse light/dark cycle (12 h/12 h) with humidity and temperature maintained at 55 ± 10% and 22 ± 2°C, respectively. SOD1 mice display a phenotype similar to human ALS symptoms, with progressive limb muscle paralysis starting around postnatal day (P)90 and reaching end‐stage ALS at around P120 (Gurney et al. 1994). NEUROSCORE tests (Hatzipetros et al. 2015) were performed for each mouse on the day of the experiment to confirm the pre‐symptomatic stage of the disease. All mice qualified for experiments had a neuroscore of 0. To investigate the impact of tsDCS on the MN electrophysiological profile, 21 SOD1 mice were randomly assigned to a two‐week tsDCS protocol, starting between P30 and P35; in vivo intracellular recordings were performed at P45–P55. Importantly, P45–P55 is considered a presymptomatic stage, just at the onset of fast‐twitch muscle fibre denervation (Hegedus et al. 2008) when functional deficits are almost undetectable (Oliván et al. 2015) but MN pathology is evident (Delestrée et al. 2014; Martínez‐Silva et al. 2018; Bączyk, Alami, et al. 2020). Importantly, at this age, the numbers of excitatory terminals on MNs from proprioceptive afferents are not reduced (Schütz 2005; Vaughan et al. 2015), however, reversible alterations to postsynaptic elements of these synapses can be detected (Bączyk, Alami, et al. 2020).

Therefore, at this time point tsDCS actions will have time to modify the disease‐affected system still containing all MN types, and will not be biased by type‐dependent cell degeneration (Pun et al. 2006; Hegedus et al. 2008).

For immunohistochemical analysis, 27 SOD1 mice were subjected to the tsDCS protocol, also starting at P30–P35. This cohort was sacrificed at P45–P55, and spinal cords were removed and processed. Following European Union recommendations, our humane endpoints were defined as an inability of the mouse to reach food or water, the loss of more than 30% of body weight over 72 h, or an inability to rise or ambulate. None of the animals used in this study reached any of the humane endpoints. All procedures performed in this study were conducted at the Department of Neurobiology of Poznań University of Physical Education (Poznań, Poland) and were approved by the Poznań Local Ethical Committee (approval number 44/2018; Poznań, Poland). All authors held valid permits for working with laboratory animals and were appropriately trained in all experimental procedures.

### 2.2 Two‐Week tsDCS to Investigate MN Synaptic Excitation Levels

TsDCS or sham polarization was applied to SOD1 G93A animals, randomly divided into Sham Control, Cathodal, and Anodal polarization groups. All groups underwent daily tsDCS or sham treatment lasting 15 min for a total of 14 days. For all groups, the tsDCS procedure started at P30–P35. To ensure adequate contact between the electrode and the skin, the mice were initially shaved on their back and abdomens. Each tsDCS session started with the administration of isoflurane anaesthesia (Isotek, Spain), initially in an animal induction chamber (Classical Vaporizer, VetFlo) using 4%–5% isoflurane in air at a delivery rate of 100–200 mL/min for 5 min. Once a medium level of anaesthesia was reached (lack of hind limb withdrawal reflex, rhythmic breathing), the mice were moved from the induction chamber and the anaesthetic was delivered through a closely fitted nose cone. Thereafter, anaesthesia was sustained with 2%–3% isoflurane in air (flow rate 100–200 mL/min). Application of a highly conductive electrolyte gel (Sigma Gel, Parker) to the animal's skin preceded the placement of the electrode to enhance electrical conductivity and prevent skin damage. A rectangular stainless steel active electrode (5 × 10 mm) was placed above the Th13‐L1 vertebrae, and a metal clip was attached to the abdominal skin flap ventral to the spinal electrode to close the polarization circuit. Polarization was applied by passing a constant current (depolarizing or hyperpolarizing) through the predescribed circuit with a custom‐made battery‐driven constant current stimulator (TP‐1, WiNUE, Poland). For anodal tsDCS, the dorsal electrode served as an anode (positive charge), whereas the crocodile clip served as a cathode (negative charge). By switching the polarity of the current source, this circuit arrangement provided immediate current reversal from anodal to cathodal and vice versa. Importantly, this approach was already confirmed to evoke depolarization or hyperpolarization of the spinal column in the segments close to the dorsal electrode from which the spinal MNs were recorded (Jankowiak et al. 2022). TsDCS was administered at an intensity of 100 μA, with either anodal or cathodal polarization applied for 15 min, producing a current density of 2 μA/mm^2^ just below the active (dorsal) electrode. The current intensity was constantly monitored to ensure that the electrical charge delivered was the same for each animal. Once the polarization was complete, the mice were moved to a cage to recover and were carefully observed for any signs of abnormal behavior. The Sham Control group received an identical anaesthesia and electrode placement protocol; however, no current was passed through the electrodes.

### 2.3 Two‐Week tsDCS to Study the Pre‐ and Postsynaptic Structures of the Ia‐MN Circuit

Sham Control, Cathodal, and Anodal animals were subjected to a two‐week tsDCS procedure identical to that described above. However, on the 10th day of the protocol, shortly after the tsDCS/Sham session, the lateral gastrocnemius (LG) muscle was bilaterally injected with cholera toxin B subunit (CTB) to retrogradely label the LG MN pool. Following the injection, the tsDCS protocol was continued for the remaining 4 days. The CTB injection procedure is briefly described in the next paragraph.

### 2.4 Retrograde Labeling of MNs

The pool of LG MNs was retrogradely labeled in all mice subjected to the two‐week tsDCS protocol (Sham Control, Cathodal, and Anodal groups) with CTB, conjugated with Alexa Fluor 555 (solution of 1.0 mg/mL CTB‐555 with clean phosphate‐buffered saline [PBS]). The animals were anaesthetised with isoflurane (2%–3% in oxygen, as described in the above tsDCS protocol) and placed on a heating pad to maintain the central body temperature at 37°C. A bilateral incision was made on the skin of the hindlimb just above the triceps surae (TS) muscle, and 5 μL CTB‐555 was injected into the LG muscle with a Hamilton microsyringe connected to a 22‐gauge needle. Following the injection, the needle was removed, the skin was sutured with sterile ligatures and a subcutaneous injection of the analgesic and anti‐inflammatory drug Meloxicam (0.4 mg/kg body weight) was given.

### 2.5 Tissue Processing for Immunohistochemistry

The day after the final polarization session (5 days after CTB injection), the animals were anesthetized with a lethal dose of Morbital (200 mg pentobarbital/kg body weight, intraperitoneal). Mice were transcardially perfused (flow 6.5 mL/min) with 40 mL 0.1 M PBS and fixed with 70 mL ice‐cold 4% paraformaldehyde (PFA). Isolated spinal cords were post‐fixed in PFA overnight at 4°C and then cryoprotected in 30% sucrose in 0.1 M PBS. The L3–L5 spinal cord segments were surrounded by Tissue‐Tek OCT cryo‐embedding compound and frozen on dry ice. Transverse sections (30 μm) were cut on the cryostat (Thermo Scientific Microm HM 520) at −19°C and collected free‐floating in cryoprotectant solution (300 mL glycerol, 500 mL 0.05 M PB pH 7.4, supplemented with 150 g sucrose) at −20°C.

### 2.6 Immunostaining of Pre‐ and Postsynaptic Structures

Free‐floating sections containing CTB + MNs (minimum six sections per animal) were washed in PBST (PBS + 0.2% Triton X‐100) and incubated in a blocking buffer (5% normal donkey serum) in PBST for 2 h at RT. Next, sections were incubated overnight at 4°C with goat anti‐VGluT1 (vesicular glutamate transporter 1; 1:500, Synaptic Systems, cat. no 135307) and rabbit anti‐GluR4 (AMPA receptor subunit 4; 1:400, D41A11, Cell Signaling, cat. no 8070) antibodies diluted with PBST. Next, sections were washed in PBST before a 2 h incubation at RT with the secondary antibodies (diluted at 1:500): donkey anti‐goat CF‐647 (Biotium 20048) and donkey anti‐rabbit Alexa Fluor 488 (Thermo Fisher Scientific 21206). Afterwards, sections were rinsed three times and mounted in ProLong Gold Antifade (Thermo Fisher Scientific). Immunofluorescence (IF) labeling was carried out in one experimental session to ensure identical conditions of tissue processing and staining.

### 2.7 Immunostaining of pCREB and Disease Marker

The free‐floating sections with CTB + MNs (minimum five sections per animal) were washed in PBST (PBS + 0.2% Triton X‐100) and incubated in a 1% blocking buffer (ab126587) for 2 h at RT. Next, sections were incubated two nights at 4°C with rabbit anti‐phospho‐CREB (Ser133) (1:200, Cell Signaling, mAb #9198), mouse anti‐misfolded SOD1 (1:500, Mèdimabs, cat. no MM‐0070‐P), and guinea pig anti‐VAChT (1:1000, Synaptic System, cat no 139105) antibodies diluted with 1% blocking buffer in PBST. Next, sections were washed in PBST, prior to 2 h incubation at RT with the secondary antibodies (diluted at 1:500): donkey anti‐rabbit Alexa Fluor 488 (#711‐545‐152), donkey anti‐mouse Alexa Fluor 647 (#715605‐150), donkey anti‐guinea pig Alexa Fluor 405 (#706‐475‐148), and donkey anti‐goat Cy3 AffiniPure (#705‐165‐003) respectively. Afterwards, sections were rinsed three times and mounted in ProLong Gold Antifade.

### 2.8 Image Acquisition and Quantification of Pre‐ and Postsynaptic Structures

Fluorescent images were acquired with an Olympus FV1200 confocal inverted microscope with a 60× DIC oil‐immersion objective (1.4 NA) and added 2.5 optical zoom. Twelve‐bit depth images of CTB‐labeled MNs, glutamate subunit 4 of the AMPA receptor (GluR4), and vesicular glutamate transporter 1 (VGluT1) consisting of digital slices were collected at 0.3 μm intervals with a pixel size of 0.0067 μm^2^. A minimum of 20 optical sections were acquired for each MN; MNs < 30 μm in diameter or displaying less than two VGluT1 + synapses were excluded from the analysis. Images were collected at constant exposure parameters. Acquisition parameters were adjusted so as to maintain fluorescence intensity from target structures below oversaturation and above underexposure.

For the morphological characterization of presynaptic (VGluT1) and postsynaptic (GluR4) structures, seven consecutive optical sections were collapsed in a maximum‐intensity projection, subjected to rolling‐ball background subtraction. Only VGluT1 + positive boutons (objects larger than 0.3 μm^2^) located in direct apposition to CTb^+^ labeling with partial signal overlap and abutting on motoneuron soma or the proximal part of a dendrite (5 μm from the soma) were selected for the analysis. Boutons larger than 10 μm^2^ were excluded from the analysis to avoid potential inclusion of aggregated synaptic terminals. Postsynaptic GluR4 structures were only selected for analysis if they colocalized with VGluT1 labeling and were located between the presynaptic terminals and the cytoplasm.

Quantification of VGluT1 and GluR4 was performed automatically using a custom macro developed in the ImageJ/Fiji software (Schneider et al. 2012). The algorithm generated binary masks based on the specific VGluT1 IF and GluR4 IF signal exceeding an intensity threshold of 800 for VGluT1 IF and 290 for GluR4 (values in 16‐bit grayscale). The detection threshold was the same for all analyzed groups and provided sufficient sensitivity and specificity to allow unequivocal discrimination of the VGluT1 boutons and GluR4 clusters in VGluT1 synapse.

Any synaptic terminal opposing a cell body was analyzed separately. The number, surface, and mean fluorescence intensity of analyzed structures were logged. The investigators performing the analysis were blinded to the origin (Anodal, Cathodal, or Sham) of the analyzed sample.

### 2.9 Image Acquisition and Quantification of pCREB and Misfolded SOD1 Levels

Confocal images were obtained by using LSM710, Axio Imager 2 microscope (Zeiss, Jena, Germany) with a Plan Apochromoat 20× air objective with 0.8 NA. Images of MNs labeled with pCREB and misfSOD1 fluorescent probes were acquired using excitation wavelengths 488 and 647 nm. The imaging of MNs localized in the L3‐L5 lumbar region labeled with VAChT. Images were collected at constant parameters of exposure, and all acquisition parameters were adjusted to maintain the emission fluorescence below oversaturation and above underexposure. All images were recorded as z‐stack and merged using maximum intensity projection mode in ImageJ/Fiji software (National Institutes of Health). For the evaluation of pCREB and misfSOD1 immunofluorescent intensity, the contour of each nucleus and MN was traced manually on the maximum‐intensity projection of confocal z‐stack in ImageJ/Fiji and saved as ROI for quantification of the mean fluorescence intensity. Similarly to the above, the investigators performing the analysis were blinded to the origin (Anodal, Cathodal, or Sham) of the analyzed sample.

### 2.10 Preparation for Electrophysiology

The methodology of electrophysiological experiments on spinal MNs in the mouse has been previously described in detail (Bączyk, Alami, et al. 2020; Jankowiak et al. 2022). Following premedication (atropine 0.20 mg/kg s.c., Polfa and methylprednisolone 0.05 mg, s.c., Pfizer), mice were anaesthetised by intraperitoneal injection of a drug cocktail (fentanyl 6.25 μg/mL (Polfa), midazolam 2.5 mg/mL (Polfa), medetomidine 0.125 mg/mL (Cp‐Pharma), injected at 10 mL/kg body weight). A suitable depth of anaesthesia was determined by the lack of hind limb withdrawal reflex. Subcutaneous needle ECG electrodes were positioned for heart rate monitoring, and the central temperature was maintained at 37°C with an infrared heating lamp and an electric blanket (TCAT‐2DF, Physiotemp). After a tracheotomy was performed, the mouse was artificially ventilated with pure oxygen (SAR‐1000 ventilator; CWE) with parameters adjusted to maintain the tidal CO_2_ level at around 4% (MicroCapstar; CWE). Right and left external jugular veins were catheterised for delivering additional doses of the anaesthetic cocktail (fentanyl 6.25 μg/mL (Polfa), midazolam 2.5 mg/mL (Polfa), medetomidine 0.125 mg/mL (Cp‐Pharma); injected at 1.7 mL/kg body weight, i.v., administered every 20–30 min) or physiological buffer (4% glucose solution containing 1% NaHCO_3_ and 14% gelatine (Tetraspan; Braun) infused at 60 μL/h). The triceps nerve (innervating the TS muscle group, including medial gastrocnemius, LG, and soleus muscles) was dissected from the surrounding tissues. Two pairs of horizontal bars (Cunningham Spinal Adaptor; Stoelting) were used to immobilize the vertebral column between the T13 and L2, and a laminectomy was made at the T13–L1 vertebrae. The dura mater was removed from the exposed L3–L4 spinal segment to allow a glass microelectrode to be inserted into the spinal cord. The exposed tissues were covered with mineral oil. At the end of the surgery, animals were paralyzed with pancuronium bromide (Pancuronium; Polfa; initial bolus of 0.1 mg, followed by additional doses of 0.01 mg every 30–40 min). From this point on, each additional dose of the anaesthetic was given at the same frequency as during the surgery, or when ECG and/or PCO_2_ levels neared the maximal physiological values.

### 2.11 Stimulation and Recording

**FIGURE 1:**
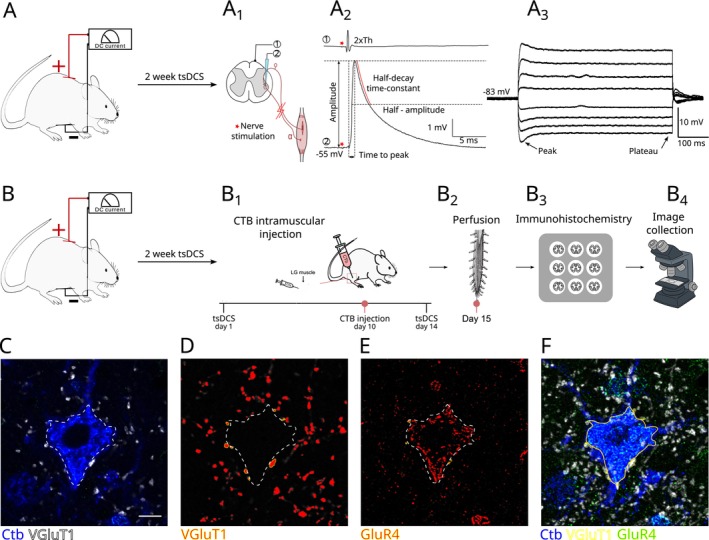
Experiment design. (A) P30–P35 SOD1 G93A mice were subjected to 2 weeks of Anodal, Cathodal, or Sham Control polarization protocol. In the drawing, the Anodal arrangement of the tsDCS electrodes is presented. One day after the last polarization session, an electrophysiological experiment was performed as outlined in (A_1_) and the Ia EPSPs (A_2_) and passive membrane properties (A_3_) of spinal MNs were investigated. (B) As in (A), P30–P35 mice were subjected to 2 weeks of Sham Control, Cathodal, or Anodal polarization; however, at the 10th day of polarization protocol (B_1_), a bilateral injection of cholera toxin B (CTB) was made into lateral gastrocnemius (LG) muscles of all animals to retrogradely label the LG MN pools. (B_2_—B_4_) 1 day after the last polarization session, mice were sacrificed, spinal cords removed, and immunohistochemical analysis of MN synaptic coverage, intracellular metabolic pathways activity, and misfolded SOD1 protein levels was performed. (C–F) Example of marko workflow for VGluT1 and GluR4 fluorescence intensity and cluster area analysis. (C) Optical section in which VGluT1‐positive presynaptic terminals were clearly visible on the CTB‐labeled motoneuron. From this reference section, a stack of ±3 consecutive optical sections (seven sections in total) is generated. (D) VGluT1‐positive presynaptic terminals are selected. To standardize their identification, an intensity threshold is applied to the VGluT1 immunofluorescence signal. The macro was programmed to generate binary masks directly from the fluorescence intensity profile, so that only pixels above the defined threshold were included. This ensured objective delineation of presynaptic terminals and eliminated user‐dependent variability. Based on these masks, the program quantified both the number of VGluT1‐positive terminals and their mean fluorescence intensity. (E) The same seven optical sections are analyzed for GluR4 immunofluorescence. A separate threshold, optimized for GluR4 labeling, was applied to generate binary masks of postsynaptic clusters. Only GluR4‐positive clusters located on the motoneuron membrane and directly apposed to VGluT1‐positive terminals were included in the analysis. Whenever additional VGluT1‐positive presynaptic terminals were identified in subsequent optical sections, they were selected and the same procedure (thresholding, mask generation, and quantification) was repeated. (F) Once all VGluT1‐positive presynaptic terminals and associated GluR4 clusters are analyzed, the outline of the motoneuron soma is manually traced based on CTb labeling. Twenty‐five micrometers scale bar in C applies to all images.

**FIGURE 2:**
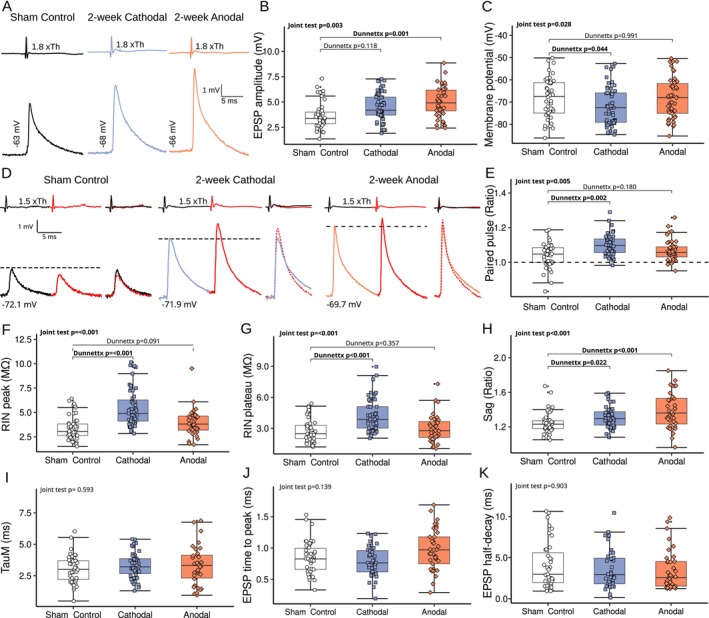
The effects of two‐week polarization protocol on SOD1 G93A mouse motoneurons electrophysiological profile. (A) Intracellular records of maximal Ia EPSPs recorded from triceps surae (TS) motoneurons (MNs) of Sham Control, Cathodal, or Anodal polarization groups. The upper trace shows a group I volley recorded from the surface of the spinal cord evoked by TS nerve electrical stimulation. The 1.8xTh (1.8xThreshold) on the upper trace corresponds to the intensity of TS nerve stimulation. The lower trace shows maximal Ia EPSPs recorded from TS MNs evoked by TS nerve stimulation. Notice a significantly higher maximal EPSP amplitude for the Anodal polarization group. (B and C) Box plots showing the data distribution from Sham Control, Cathodal and Anodal polarization groups for maximal Ia EPSPs and resting membrane potential respectively. (D) Intracellular recordings of MNs subjected to the paired‐pulse protocol from Sham Control, Cathodal, and Anodal polarization groups. Panel description as in (A) For each test, the second response is marked red and superimposed as a dotted line on the first response to the right of the original trace. A dashed line indicates the 1st response amplitude. (E, as in B and C) But showing the paired‐pulse protocol results with the dashed line showing the border between paired‐pulse depression and paired‐pulse facilitation. Notice a paired‐pulse depression in a large subset of MNs in the Sham Control group. This phenomenon is largely reduced in the Cathodal polarization group with only a single MN showing paired‐pulse depression. (F–K) Box plots showing data distribution of peak (F) and plateau (G) input resistance, SAG ratio (H), membrane time constant (I), EPSP time to peak (J), and EPSP half‐decay time constant (K). Each point on the boxplot indicates a single MN. The lower and upper hinges correspond to the first and third quartiles (25th and 75th percentiles, respectively). The upper whisker extends from the hinge to the largest value no further than 1.5× the interquartile range (IQR). The lower whisker extends from the hinge to the smallest value, at most 1.5× the IQR. Outliers were checked for correctness and are plotted individually if they met all inclusion criteria. The significant differences between the investigated groups are marked in bold at *p* < 0.05.

MN electrophysiological properties were measured with glass micropipettes (tip diameter 1.0–1.5 μm, impedance 20–30 MΩ) filled with a mix of 2 M K‐acetate and 0.1 M QX‐314 (sodium channel blocker; Sigma‐Aldrich). Intracellular recordings were obtained with an Axoclamp 900A amplifier (Molecular Devices) connected to a Power1401 interface (CED, sampling rate 20 kHz) operated by Spike2 software (CED). The amplifier system was used in bridge mode to record excitatory postsynaptic potentials (EPSPs; single response or paired‐pulse (PP) responses) or in discontinuous current clamp (DCC) mode (switching rate 7–8 kHz) to record the responses to square pulses necessary to determine input resistance. Peripheral stimulation of the triceps nerve enabled us to identify MNs based on an “all‐or‐nothing” antidromic action potential. This method of MN antidromic identification was still possible since it takes 30–90 s for the QX‐314 to diffuse from the electrode tip to the cell body and block the antidromic spike. The TS nerve was stimulated with constant current pulses of 0.1 ms duration and amplitude up to 50 μA delivered at 3 Hz (DS4, Digitimer), but not more than 2.5 × threshold (Th) to activate the most excitable fibres in the nerve. An electrode positioned at the dorsal surface of the spinal cord was used to record the group I afferent volley in response to peripheral nerve stimulation (Figure 1A_1_). All the MNs selected for the analysis were characterized by a resting membrane potential (RMP) more hyperpolarised than −50 mV and an initial overshooting action potential. The maximal EPSP amplitudes were measured from the RMP (measured just after the stimulation artefact) to the most depolarised part of the voltage response (Figure 1A_2_) and EPSP time to peak was measured from the beginning of EPSP to the maximal amplitude. The EPSP half decay time constant was measured by plotting an exponential curve to the descending part of the EPSP trace, to the point when it decayed 50% of the maximal EPSP amplitude (Figure 1A_2_). In the PP protocol, the peripheral nerve was stimulated with a pair of identical constant‐current stimuli of 0.1 ms duration separated by 10 ms intervals that elicited EPSPs (Figure 2D). The PP ratio was calculated by measuring the amplitude of the second EPSP measured from the most hyperpolarised part of the voltage trace after the first EPSP from the pair, and dividing it by the amplitude of the first EPSP measured from the RMP. This method of PP analysis is independent of possible temporal summation of the EPSP traces, which can occur if the EPSP decay is especially long (Jiang and Abrams 1998; Zhang and Schneider 2011; Jackman et al. 2016). The PP ratio was measured for each individual EPSP pair and then averaged across the recording period (at least 10 EPSP pairs per average). The MN input resistance at RMP (RIN) was calculated by measuring the membrane voltage deflection in response to a series of small‐amplitude square current pulses (−2 to +2 nA, 500 ms), as described previously (Manuel et al. 2009, and outlined in Figure 1A_3_). Injecting small hyperpolarizing current pulses (−5 nA, 1 ms) enabled us to measure the membrane time constant on the relaxation of the membrane potential (TauM). At the end of the experiment, animals were euthanised with a lethal intravenous dose of sodium pentobarbital (200 mg/kg).

### 2.12 Statistics

All plots and statistical analyses were programmed in RStudio 2023.06.1 (Posit Software, PBC) with appropriate libraries. Plots were created with the ggplot2 package (Wickham 2016). Generalized Linear Mixed Models (GLMM) were used to determine the significance level of our findings. Due to the high variability of MN electrophysiological properties in a single animal (Binder and Powers 1999; Powers et al. 2000; Manuel and Zytnicki 2011), MNs recorded from individual animals were pooled together to form the Sham Control, Cathodal, and Anodal groups. We first visualized the data spread (by plotting the data range across three conditions) and performed descriptive statistics and normality testing (Shapiro–Wilk test) to select a family parameter (Gaussian, Binomial, Poisson, etc.) for our model. Importantly, the GLMM models do not require normal distribution of the data, as they introduce the “link” function to the selected data family which transforms the expected value to a continuous scale for linear modeling. Indeed, the a priori data normalization is not recommended for GLMM models (Lo and Andrews 2015; Salinas Ruíz et al. 2023).

The model was plotted using the glmmTMB package (Brooks et al. 2017) which allowed us to correct for zero‐inflation and data dispersion. The fixed effect factor for the model was the polarization intervention (Sham Control, Cathodal, or Anodal). Introducing the random factor to the model (Mouse), allowed us to compensate for the dependence of MN properties on the analyzed animal often seen in in vivo electrophysiological investigations (Highlander et al. 2020) and sample inflation from recording multiple MNs from a single animal. To validate the fitted model, a Residual Diagnostics for HierArchical (Multi‐level/Mixed) Regression Model was performed using the DHARMa package. A plot of the scaled residuals was created by simulating from the fitted model, and data deviation, dispersion, and variance were assessed. If a significant deviation from the model was found, then the dispersion correction or data family correction was introduced and the model was refitted to meet all assumptions. Only the models that met all assumptions were used for further significance analysis. The significance of the fixed effect was established with joint tests of the terms in a model, and between‐group significance was then assessed with contrasts of the estimated marginal means for specified factors from the emmeans package (Lenth 2023), with the Dunnettx adjustment method for comparing a family of two estimates. The significance level of the fixed effect was set at *p* < 0.05, and the Cohen's (*d*) effect size was calculated. Post hoc power calculations were not performed, due to their uninformative nature (Goodman and Berlin 1994; Hoenig and Heisey 2001; Althouse 2021; Heckman et al. 2022; Heinsberg and Weeks 2022). For each comparison, we report the Estimated Marginal Mean value±SE, significance level, and Cohen's effect size ± SE. In the box plots, each data point represents a single MN for electrophysiological data, and a single synapse or a single MN for immunohistochemical data.

## 3 Results

### 3.1 Two Weeks of Anodal tsDCS Significantly Increases EPSP Amplitudes in SOD1 Mice

In the first series of experiments, we investigated whether two‐week tsDCS triggers MN plasticity, measured as a change in the levels of MN synaptic excitation by Ia afferents. The analysis was performed on *n* = 47 MNs in the Sham Control group (*n* = 5 mice), *n* = 52 MNs in the Cathodal group (*n* = 10 mice), and *n* = 42 MNs in the Anodal polarization group (*n* = 6 mice). Figure 2 shows that anodal polarization repeated 15 min daily for 2 weeks significantly increased the maximal Ia EPSP amplitudes recorded from TS MNs of SOD1 G93A mice. The maximal amplitudes of unitary Ia EPSPs were on average 40% larger in the Anodal polarization group when compared to the Sham Control group (5.30 ± 0.36 mV vs. 3.78 ± 0.28 mV, Joint test *F*(2, inf) = 5.734, *p* = 0.003, post hoc Dunnettx *p* = 0.001, *d* = 1.21 ± 0.36, Figure 2A,B). This increase of the EPSP amplitude was not followed by changes in the MNs' passive membrane properties (Figure 2C,F–I, all tests *p* > 0.05), except for SAG ratio, which was significantly increased (from 1.31 ± 0.02 in Sham Control to 1.41 ± 0.04 in Anodal group, Joint test *F*(2, 123) = 8.144, *p* = 0.001, post hoc Dunnettx *p* < 0.001, *d* = 1.29 ± 0.34). In contrast, no change in maximal EPSP amplitude was seen in the Cathodal polarization group (post hoc *p* = 0.118, Figure 2A,B). However, 2‐week cathodal tsDCS application resulted in a strong hyperpolarization of cells RMP (from −68.2 ± 1.28 mV in Sham Control to −72.1 ± 1.19 mV in Cathodal group, Joint test *F*(2, 138) = 3.677, *p* = 0.028, post hoc *p* = 0.044, *d* = 0.46 ± 0.20), increase of peak (from 3.32 ± 0.15 MΩ to 5.47 ± 0.25 MΩ, Joint test *F*(2, inf) = 31.421, *p* < 0.001, post hoc Dunnettx *p* < 0.001, *d* = 1.56 ± 0.22, Figure 2F) and plateau input resistance (from 2.71 ± 0.15 MΩ to 4.26 ± 0.23 MΩ, Joint test *F*(2, inf) = 18.861, *p* < 0.001, post hoc Dunnettx *p* = 0.001, *d* = 1.10 ± 0.22, Figure 2G), and an increase of the SAG ratio (from 1.23 ± 0.02 to 1.31 ± 0.02, Joint test *F*(2, 123) = 8.144, *p* < 0.001, post hoc Dunnettx *p* = 0.022, *d* = 0.68 ± 0.29, Figure 2H). Interestingly, further analysis showed that cathodal polarization significantly increased the PP ratio (from 1.04 ± 0.01 to 1.10 ± 0.01, Joint test *F*(2, inf) = 5.258, *p* = 0.005, post hoc Dunnettx *p* = 0.002, *d* = 0.95 ± 0.25, Figure 2D,E). This is in sharp contrast to the multiple cases of pathological PP depression seen in the Sham Control group, as we have previously reported for SOD1 animals (Bączyk, Alami, et al. 2020), (Figure 2D,E).

These results point toward the synaptic origin of the Ia EPSP amplitude increase following tsDCS. Anodal tsDCS increases the maximal EPSP amplitudes while not affecting MN passive membrane properties.

In the lack of changes to MN membrane time constant and passive input resistance following anodal tsDCS, we analyzed the time course of EPSPs to determine possible alterations of AMPA receptor dynamics. Both maximal EPSP rise time and the maximal EPSP half‐decay time constant were not significantly different between the studied groups (Joint test *F*(2, 116) = 2.006, *p* = 0.139 and Joint test *F*(2, inf) = 0.102, *p* = 0.204 for EPSP half‐decay time and EPSP rise time respectively, Figure 2J,K) indicating that alterations to AMPA receptor activation/deactivation do not contribute to the observed Ia EPSP increase.

### 3.2 Two Weeks of tsDCS Significantly Affects Ia Synapse Morphology

Having identified the impact of tsDCS on MNs' electrophysiological profile and synaptic excitation levels, we set out to determine if the observed changes can be linked to morphological changes in the Ia synapse. We first quantified the level of fluorescence intensity of Ia synaptic boutons, identified by the presence of VGluT1 on the soma of CTB‐labeled LG MNs in spinal cords harvested from mice subjected to the Sham Control (*n* = 5), Cathodal (*n* = 5) or Anodal (*n* = 5) polarization protocol. Importantly, while all proprioceptive afferents express VGluT1 (and not VGluT2), only the Ia afferents project to spinal MNs; therefore, the VGluT1 boutons on MNs can be ascribed to Ia terminals. While in rats a limited number of VGluT1 contacts in laminae IX can arise from the cortico‐spinal tract (CST Persson et al. 2006a), the monosynaptic CST contacts to spinal MNs basically do not exist in mice (Alstermark and Ogawa 2004; Alstermark et al. 2004).

**FIGURE 3:**
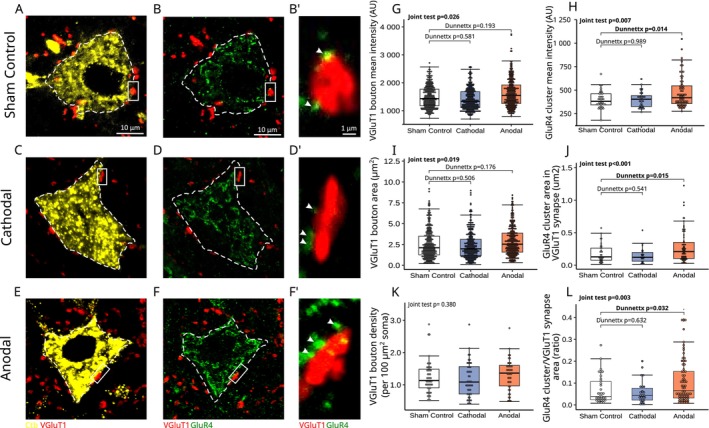
VGluT1 and GluR4 response to 2 weeks tsDCS. (A–F) tsDCS‐dependent changes in VGluT1 and GluR4 fluorescence intensity (AU) and area (μm^2^) across three experimental conditions: Sham Control (A, B), Cathodal (C, D), and Anodal (E, F) polarization, respectively. Each large panel depicts a single motoneuron (MN), with white dashed outlines indicating the approximate cell body shape (scale bar: 10 μm). A magnified region (B′, D′, F′) illustrates the VGluT1 synapse contact on the MN cell body with white arrows marking the GluR4 cluster juxtaposed to the presynaptic VGluT1 bouton (scale bar: 1 μm). (G, H), The box plots display the data distribution of VGluT1 boutons and GluR4 clusters fluorescence intensity (AU). (I, J), Similar to (G, H), but displaying data distribution for VGluT1 bouton and GluR4 cluster contact area. (K), Data distribution for the density of VGluT1 synaptic contacts on MN soma. (L), Data distribution for Glur4 cluster area vs. its respective VGluT1 bouton area. Notice a significantly higher fluorescence intensity, area, and ratio of GLuR4 clusters when compared to the Sham Control group. Boxplot and statistical description as in Figure 2.

Figure 3 shows no significant effect of two types of polarization on Ia monosynaptic terminals on spinal MNs. The level of fluorescence intensity of VGluT1 boutons on LG MNs was not significantly different between both polarization groups and the Sham Control group (Joint test, F(2, inf) = 3.651, *p* = 0.026, post hoc Dunnettx *p* = 0.192, and *p* = 0.581 for Anodal and Cathodal polarization groups, respectively, Figure 3A,C,E,G). A similar lack of significant effect was seen for the average VGluT1 bouton size, with no significant difference when either group was compared to Sham Control (Joint test, *F*(2, inf) = 3.983, *p* = 0.019, post hoc Dunnettx *p* = 0.176 and *p* = 0.506 for Anodal and Cathodal polarization, respectively, Figure 3A,C,E,I. Finally, the MN soma size (Joint test *F*(2, inf) = 0.060, *p* = 0.942) and density of VGluT1 + synapses on the MN membrane (Joint test *F*(2, inf) = 0.968, *p* = 0.380, Figure 3K) were both not affected by either polarization protocol indicating that tsDCS did not cause Ia synaptic sprouting.

Since we did not find any significant changes in the number and size of the VGluT1 synaptic contacts on spinal MNs upon tsDCS intervention, we then asked if alterations to the postsynaptic elements of the Ia synapse could explain the electrophysiological data. To this end, we quantified the level of fluorescence intensity of the GluR4 subunit of the AMPA receptor, as our previous investigations linked their lower expression to reduced maximal EPSP amplitudes in SOD1 animals (Bączyk, Alami, et al. 2020). Importantly, GluR4 is recognized as the principal post‐synaptic subunit of the AMPA receptors at VGluT1 synapses in MNs (Ragnarson et al. 2003). We were able to detect a significant increase in the GluR4 IF signal intensity following the anodal polarization protocol (from 400 ± 17.4 AU to 461 ± 14.4 AU, Joint test *F*(2, inf) = 4.926, *p* = 0.007, post hoc Dunnettx *p* = 0.014, *d* = 0.56 ± 0.21, Figure 3B,D,F,H), whereas no change was observed following the cathodal polarization protocol (post hoc Dunnettx *p* = 0.990, Figure 3H). Further analysis showed that the area of the GluR4 was also significantly larger in the Anodal than in the Sham Control group (0.280 ± 0.02 μm2 vs. 0.180 ± 0.02 μm2, Joint test *F*(2, inf) = 7.808, *p* < 0.001, post hoc Dunnettx *p* = 0.015, d = 0.55 ± 0.21), while no significant effect of the Cathodal polarization on the GluR4 cluster area was detected (post hoc Dunnettx *p* = 0.541, Figure 3J,L). These results consistently show that the anodal polarization triggered strong plasticity of the Ia synapse, expressed in the recovery of GluR4 subunits of the AMPA receptor which can explain the increase in Ia EPSPs amplitude following anodal tsDCS.

### 3.3 Two Weeks of tsDCS Does Not Change Activity‐Dependent Intracellular Pathways Converging on CREB and Cellular Disease Marker Levels

**FIGURE 4:**
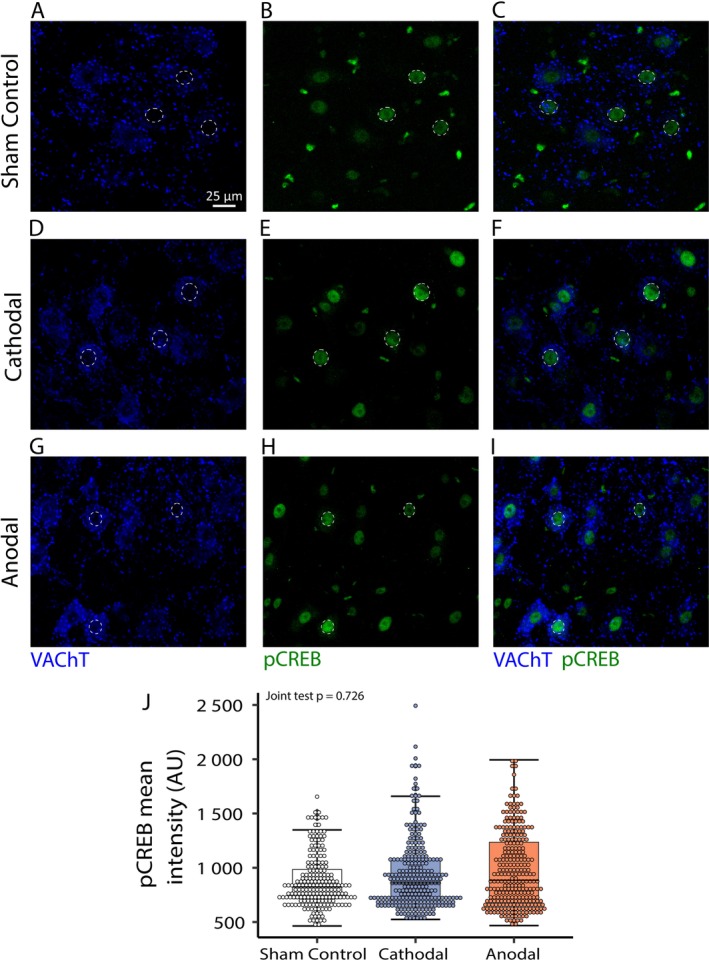
Lack of tsDCS effects on pCREB levels. (A–I) Modest tsDCS‐dependent changes in pCREB fluorescence levels (AU) of L3–L4 motoneurons in Sham Control (A–C), Cathodal (D–F), and Anodal (G–I) polarization groups. The dashed outlines indicate the approximate shape of MN nuclei in which the pCREB levels were measured (scale bar: 25 μm). (J) The box plot displays the data distribution of pCREB fluorescence intensity (AU) across three experimental conditions: Sham Control, Cathodal, and Anodal polarization groups, respectively. Note a lack of significant effect of tsDCS on pCREB fluorescence intensity levels. Boxplot and statistical description as in Figure 3.

**FIGURE 5:**
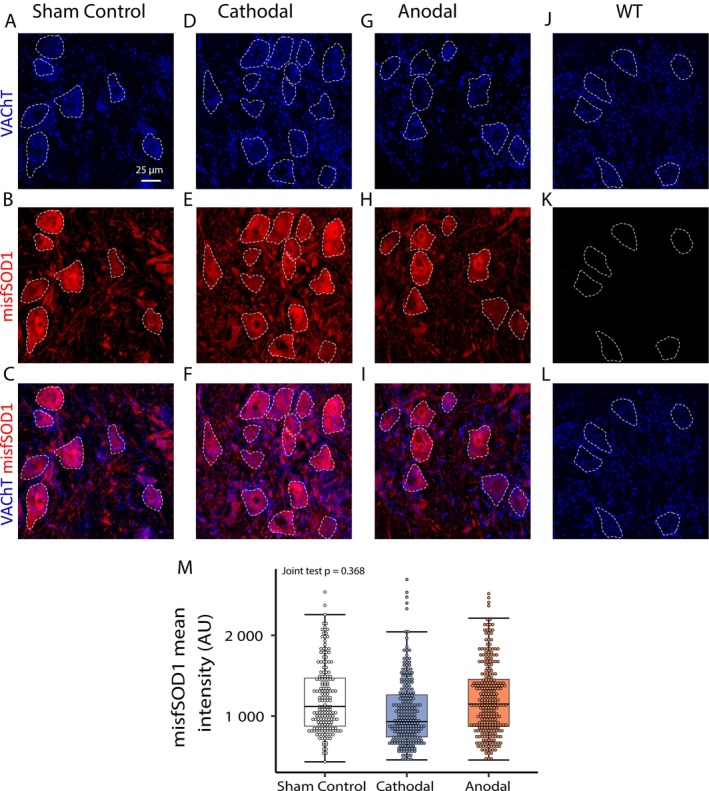
Lack of tsDCS effects on misfSOD1 levels. (A–I) Modest tsDCS‐dependent changes in misfSOD1 fluorescence levels (AU) of L3–L4 motoneurons in Sham Control (A**–**C) Cathodal (D**–**F), and Anodal (G**–**I) polarization groups. (J**–**L) Negative control for misfSOD1 antibody, showing no labeling in WT MNs. The dashed outlines indicate the approximate cell body shape (scale bar: 25 μm). (M) The box plot displays the data distribution of misfSOD1 fluorescence intensity (AU) across three experimental conditions: Sham Control, Cathodal, and Anodal polarization groups, respectively. Note a lack of significant effect of tsDCS on misfSOD1 fluorescence intensity levels. Boxplot and statistical description as in Figure 3.

Our previous work showed that a chronic increase of MN synaptic excitation levels induced by chemogenetics ameliorates disease burden, as indicated by a reduction of misfolded SOD1 protein levels (Bączyk, Alami, et al. 2020). These experiments showed a similar, strong increase of MN synaptic excitation following two weeks of anodal polarization. In parallel, a strong increase in input resistance was seen following cathodal polarization (Figure 2). Both of these effects may translate into stronger MN activity (Manuel and Zytnicki 2011), which in turn can activate intracellular pathways and provide activity‐dependent neuroprotection (Bączyk, Alami, et al. 2020). Therefore, in the final set of experiments, we set out to investigate whether the observed tsDCS effects translate into changes in intracellular pathway activity and disease marker levels. In opposition to our expectations, tsDCS had a negligible effect on the analyzed parameters. Figure 4 shows that indeed, both cathodal and anodal polarization failed to produce a significant change in the levels of phosphorylated pCREB, a major marker of activity‐dependent intracellular pathways activity (Joint test *F*(2, inf) = 0.31, *p* = 0.726). Similarly, Figure 5, shows no significant effect of tsDCS on the levels of an intracellular disease marker—the misfolded SOD1 protein (Joint test *F*(2, inf) = 0.999, *p* = 368). These results indicate that while anodal tsDCS induces strong neuroplasticity of the Ia synapse, it fails to provide activity‐dependent neuroprotection to ALS MNs.

## 4 Discussion

This study is the first to directly investigate the impact of a two‐week tsDCS protocol applied to SOD1 mice at a presymptomatic stage of ALS on the electrophysiological and biochemical properties of MNs. We show a strong polarity‐dependent effect of tsDCS on Ia EPSPs and postsynaptic elements of the Ia synapse in SOD1 mouse MNs. The effects of anodal tsDCS are predominantly facilitatory and include an increase of maximal Ia EPSP amplitude and an increase in the area of the GluR4 subunit of the AMPA receptor. On the other hand, the effects of cathodal polarization are modest in their effect on EPSPs but show a strong increase in MN input resistance and an increase of the PP ratio. While the effects of anodal polarization on the spinal MN electrophysiological profile and Ia synapse morphology are apparent, they do not translate into significant alterations of the intracellular activity‐dependent metabolic pathway converging on CREB, and they do not modify the cellular disease burden.

### 4.1 The Effects of tsDCS Are Similar Between Acute and 2‐Week Application

The effects of the 2‐week tsDCS protocol on spinal MN physiology in SOD1 mice largely reflect the acute effects of a single tsDCS application. In this study, 2‐week anodal tsDCS significantly increased Ia EPSP amplitude (Figure 2), while at the same time, it did not affect the MN passive membrane properties. We have previously shown that acute anodal tsDCS transiently increased the Ia EPSP amplitude up to 1 h after tsDCS application and did not provoke long‐term changes in the passive membrane properties (Jankowiak et al. 2022). However, while the acute effects of anodal tsDCS are likely a result of an increase in Ia afferent excitability (Jankowiak et al. 2022), the effects of 2‐week tsDCS may reflect neuroplasticity at the Ia‐MN synapse. This is supported by the fact that in the acute setting, anodal tsDCS increased Ia EPSPs amplitudes only during the first hour after tsDCS application and therefore could only be caused by a transient increase in Ia fibre excitability or a transient alteration of the Ia synapse activity. In the current investigation, MN recordings were performed 1 day after the last tsDCS session, i.e., long after any prolonged effects of the single tsDCS session were detectable (Jankowiak et al. 2022), and therefore, the observed EPSP increase cannot be linked to the acute tsDCS effects.

The case is similar for the effects of 2‐week cathodal tsDCS on MN passive membrane properties. Indeed, the increase of peak and plateau RIN, upregulation of SAG and hyperpolarization of RMP is seen after acute cathodal tsDCS application, but it is not visible already 15 min after switching the DC current off (Jankowiak et al. 2022). In the current investigation, alterations to RIN and RMP were seen more than 24 h after the last tsDCS session and therefore cannot be explained by the sustained effects of the acute cathodal tsDCS.

### 4.2 The Increase in Ia EPSP Amplitude Is Not Caused by Alterations of MN Membrane Properties

The average amplitude of maximal EPSPs in the Anodal polarization group was significantly larger than in the Sham Control or Cathodal groups, and indeed very similar to maximal Ia EPSPs in untreated WT mice as presented in (Jankowiak et al. 2022). This increase may be caused by either cell‐autonomous mechanisms acting on the cell's membrane properties, such as input resistance (Manuel and Zytnicki 2011), RMP (Eccles et al. 1962; Edwards et al. 1976), activity of several types of inward and outward ion currents (PICs, Ih current), (George et al. 2009; Sharples and Miles 2021), and number of available AMPA receptors at the Ia synapse (Burke et al. 1988; Montes et al. 2015); or by extracellular factors such as an increase in Ia synapse number and density (Lenoir et al. 2018), or the levels of available neurotransmitter at presynaptic release sites. Due to our experimental setup utilizing electrical stimulation of Ia afferents, we can exclude a possible contribution of altered muscle spindle activation that could otherwise impact the EPSP amplitude. Our results indicate that it is also unlikely that the increase in maximal Ia EPSP amplitude in the Anodal polarization group was caused by the alteration of passive membrane properties, as the cell's membrane potential, time constant, and input resistance were not significantly affected. This is further confirmed by a lack of increase in maximal EPSP amplitude in the Cathodal polarization group, in which we found a strong increase in the peak and plateau input resistance that should classically result in increased EPSP amplitude (Manuel and Zytnicki 2011). Finally, it is also unlikely that the observed effects result from the increased activity of the PICs or Ih current. While the increase in the SAG ratio may indicate an increase in the Ih current, this is classically linked to inhibition of the cell's synaptic excitation and therefore cannot explain the EPSP increase (George et al. 2009). In the case of the PICs, it is well known that PICs amplify the stationary (vibration‐induced) synaptic input to spinal MNs (Lee and Heckman 2000). However, the time‐course of the electrically induced EPSPs is too fast for the calcium PICs to be activated. On the other hand, the sodium PICs are much faster and may have an impact on the electrically induced EPSPs (Manuel et al. 2007), especially as they are activated close to the MN firing threshold (Harvey et al. 2006). However, the use of QX‐314, which blocks sodium and calcium currents during the recording session, limits the possible contribution of PICs to the observed phenomenon. It therefore seems that MN membrane properties play only a minor role in the tsDCS‐induced alterations of EPSP amplitudes.

### 4.3 Anodal tsDCS Provokes Remodeling of the GluR4 Subunit of the AMPA Receptor at the Ia Synapse, Thus Explaining the Increase of Ia EPSP Amplitude

The intensity of the fluorescence signal and the area of the GluR4 subunits of the AMPA receptor were significantly increased in LG MNs of SOD1 mice following 2 weeks of anodal tsDCS. This indicates that more neurotransmitter binding sites were available at the Ia synapse, thus allowing more effective EPSP generation. Taken together with the lack of changes in the VGluT1 labeling, this morphological alteration can well explain the increased Ia EPSP amplitudes following anodal tsDCS.

This remodeling of the postsynaptic elements of the Ia synapse is likely a consequence of increased excitability of the Ia afferent fibers, resulting in stronger synaptic input to spinal MNs. Importantly, an increase in the levels of GLuR4 subunits of the AMPA receptors was seen both after chemogenetic upregulation of the activity‐dependent PKA pathway in spinal MNs (Bączyk, Alami, et al. 2020) and after an increase of abducens nerve motor nuclei activity following eye‐blink conditioning (Keifer 2001; Keifer and Clark 2003). In our previous studies, we have shown that a single 15‐min session of anodal tsDCS provokes long‐term increases in Ia afferent activity (Jankowiak et al. 2022). Similarly, the works of Jankowska and Hammar (Jankowska and Hammar 2021) show that tsDCS‐induced afferent neuromodulation can last up to 2 h following treatment. Therefore, it may be expected that the excitability of Ia afferents in tsDCS‐treated SOD1 mice would last long after the end of the tsDCS session and trigger adaptive changes in Ia synapses on spinal MNs.

### 4.4 Two Weeks of tsDCS Has a Neglective Impact on the Activity of Intracellular Pathways Converging on CREB and Cellular Disease Markers

Chronic activation of the PKA pathway in SOD1 animals leads to an increase in MN synaptic excitation and a decrease of misfolded SOD1 protein in spinal MNs (Bączyk, Alami, et al. 2020). As in our experiments, a significant increase in Ia EPSP amplitude was seen following two weeks of anodal tsDCS, we expected that this would provoke an increase in the activation of intracellular metabolic pathways converging on CREB and a reduction of misfolded SOD1 protein levels. However, this was not the case, as levels of pCREB and misfSOD1 were not affected by any type of polarization. This discrepancy is likely caused by different interventions used to increase MN synaptic excitation. In Bączyk, Alami, et al. (2020) an increased Ia excitation of MNs was secondary to an upregulation of PKA pathway activity by ionophoretic injections of cAMP into recorded MN, or by the chemogenetic DREED technique, chronically increasing PKA activity. Both these techniques work downstream of MN synaptic excitation and actually do not require an increased MN activation (Bączyk, Alami, et al. 2020). With tsDCS, the most logical action sites are the primary afferents traversing the dorsal spinal cord (Jankowska and Hammar 2021). Increased afferent excitability allows for more efficient MN synaptic excitation and may trigger activity‐dependent molecular adaptations which should be detectable by altered pCREB levels. While the increase of EPSP amplitudes following anodal tsDCS supports the hypothesis of stronger MN synaptic excitation, it does not trigger the expected cellular response. This points to a fundamental “decoupling” in the transmission of synaptic signals to intracellular pathways. Indeed, these results are consistent with a recent report of no increase in pCREB levels following increased MN Ia synaptic excitation triggered with an AMPA receptor agonist (Grycz et al. 2025). As the reduction of misfSOD1 is critically dependent on the increase in pCREB levels (Bączyk, Alami, et al. 2020), the lack of this increase by tsDCS is a likely cause of the negligible effect of our intervention on misfolded SOD1 levels.

### 4.5 Changes in the PP Ratio Point to Differential Effects of Anodal and Cathodal Polarization

We found that the PP ratio was significantly increased by cathodal polarization, while anodal polarization had only a modest effect on this parameter. This is in line with our previous data showing a decrease in the PP ratio following a single anodal tsDCS session when increased Ia afferent activity oversaturates the limited number of available AMPA receptors (unpublished results). In this investigation, the reduction of the PP ratio was prevented by the recovery of the GluR4 subunits of the AMPA receptors, therefore pointing to the synaptic location of the anodal tsDCS effects. As the cathodal tsDCS failed to produce any alterations in Ia synapse morphology and Ia EPSP properties, the increase of the PP ratio following cathodal tsDCS may, on the other hand, reflect tsDCS action of cells' input resistance, allowing more effective EPSP generation even in the presence of low AMPA receptor number.

### 4.6 Anodal vs. Cathodal tsDCS

The difference between anodal and cathodal tsDCS effects on spinal circuits' excitability is non‐unequivocal. Several reports in rats indicate that cathodal tsDCS increases spinal circuits' output by facilitating myelinated afferents excitability (Jankowska et al. 2017) and synaptic transmission (Bolzoni and Jankowska 2015), while at the same time it has a negligible effect on MN excitability (Bączyk et al. 2019; Bączyk, Drzymała‐Celichowska, et al. 2020b). On the other hand, in rats anodal tsDCS was found to increase MN intrinsic excitability (Bączyk et al. 2019), but had a depressive impact on the afferents (Bolzoni and Jankowska 2015; Jankowska et al. 2016). Similar discrepancies exist in mouse models where both facilitatory (Ahmed 2014a; Jankowiak et al. 2022) and inhibitory (Ahmed 2014b; Mekhael et al. 2019) effects of anodal tsDCS were observed. A recent modeling study suggested that the electrical field orientation rather than the current polarity plays an essential role in facilitatory vs. inhibitory effects of tsDCS (Gigliotti and Pereira 2025). This is supported by a well‐known differential effect of radial and transverse electrical fields on neuronal excitability (Rahman et al. 2013; Lafon et al. 2017). While in our investigations a majority of the synaptic excitatory effects were connected to anodal tsDCS, we cannot exclude that increased input resistance following cathodal tsDCS may contribute to increased MN intrinsic excitability. However, the unequivocal distinction between anodal and cathodal tsDCS on MN excitability is still not available.

### 4.7 Study Limitations

All care was taken to standardize the conditions for each experiment. However, some factors potentially impacting the effects of the tsDCS application could not be avoided. The most important would be the inability to directly predict the spatial orientation of recorded neurons with respect to the applied electrical field. It has already been established in both in vitro experiments (Rahman et al. 2013; Lafon et al. 2017) and modeling studies (Rattay 1999; Elbasiouny and Mushahwar 2007) that radial and transverse electrical fields have different impacts on neuronal compartments depending on their spatial orientation. While spinal MN somas are located in the Rexed lamina IX, the direct orientation of the axon initial segment and dendritic arborization can vary between individual neurons (see Figures 3, 4, 5). Therefore, some neurons might be preferentially affected by tsDCS.

Next, our investigations were confined to MNs located in the lumbar spinal cord. However, there is a significant difference between the rate of motor pool degeneration in ALS: pools innervating muscles containing predominantly fast muscle fibres degenerate faster than those composed mainly of slow muscle fibres. It is possible that tsDCS has differential effects on flexor and extensor MNs, especially in the framework of different synaptic inputs to these motor pools.

Further, 2‐week tsDCS was applied under isoflurane anesthesia which could have affected PICs and MN excitability. Similarly, while the surgical anesthesia used during EPSP recording does not suppress the spinal reflexes and Ia afferent excitability (Wang et al. 1992; Saberfard et al. 2022), we cannot exclude the possibility that reduced descending monoaminergic drive affected MN electrophysiological profile.

Finally, as current flows between the tsDCS electrodes, the tissues directly below the electrodes can experience a pH shift (negative pH shift close to Anode and positive pH shift close to Cathode). Although this shift most likely receded during the 24 h that separated the last tsDCS season from the in vivo experiments, we cannot exclude the possibility that repeated pH change plays a role in the observer MN neuroplasticity (Ruffin et al. 2014).

## 5 Conclusions

This study is the first to demonstrate a pronounced polarity‐dependent plasticity of Ia proprioceptive synapses on spinal MNs in the SOD1 G93A mouse model of ALS following a two‐week tsDCS.

Anodal (depolarizing) tsDCS significantly enhances MN synaptic excitation and restores the postsynaptic components of the Ia proprioceptive synapse. In contrast, cathodal (hyperpolarizing) tsDCS markedly increases MN input resistance but does not affect Ia synapse morphology. Despite the strong neuromodulatory effects, both anodal and cathodal tsDCS fail to significantly alter the cellular burden of the disease.

## Author Contributions

**T. Jankowiak:** conceptualization, data curation, formal analysis, investigation, methodology, software, validation, visualization, writing – original draft, writing – review and editing. **M. Cholewiński:** data curation, formal analysis, investigation, visualization, writing – original draft, writing – review and editing. **K. Kryściak:** data curation, investigation, writing – original draft, writing – review and editing. **E. Krok:** data curation, formal analysis, methodology, resources, writing – original draft, writing – review and editing. **K. Grycz:** data curation, formal analysis, investigation, methodology, software, validation, visualization, writing – original draft, writing – review and editing. **M. Bączyk:** conceptualization, data curation, formal analysis, funding acquisition, investigation, methodology, project administration, resources, software, supervision, validation, visualization, writing – original draft, writing – review and editing.

## Funding

This work was supported by the National Science Center grants no. 2017/26/D/NZ7/00728 and 2019/35/B/NZ4/02058 granted to M. Bączyk.

## Conflicts of Interest

The authors declare no conflicts of interest.

## Data Availability

All data supporting the results of this study are included in the manuscript. Electrophysiological and immunohistochemical datasets and custom‐made scripts for data analysis are provided on a public access repository Zenodo DOI: https://doi.org/10.5281/zenodo.17513623. All raw recordings are stored in the private data repository at https://box.pionier.net.pl/ provided by the Poznan Supercomputing and Networking Center affiliated with the Institute of Bioorganic Chemistry of the Polish Academy of Sciences. Access to the raw data can be provided upon request.
